# Dynamic Modeling and Structural Optimization of a Partially Laminated Piezoelectric–Metal–Piezoelectric Actuator

**DOI:** 10.3390/nano16090517

**Published:** 2026-04-25

**Authors:** Xingen Kuang, Cuiying Fan, Zhi Li, Guoshuai Qin, Minghao Zhao, Chunsheng Lu

**Affiliations:** 1 School of Mechanics and Safety Engineering, Zhengzhou University, Zhengzhou 450001, China; xingenkuang@gs.zzu.edu.cn (X.K.); memhzhao@zzu.edu.cn (M.Z.); 2 School of Electromechanical Engineering, Henan University of Technology, Zhengzhou 450001, China; gsqin@haut.edu.cn; 3 School of Civil and Mechanical Engineering, Curtin University, Perth, WA 6845, Australia; c.lu@curtin.edu.au

**Keywords:** piezoelectric actuator, piezoelectric–metal–piezoelectric laminated beam, natural frequency, output displacement, analytical solution

## Abstract

Piezoelectric actuators are core components in precision motion control due to their unique electromechanical coupling properties. This paper establishes a dynamic model for a partially laminated piezoelectric–metal–piezoelectric beam actuator based on the Euler–Bernoulli beam theory. The model comprises symmetrically bonded piezoelectric layers on both sides of a central metal substrate, with the piezoelectric material partially distributed along the beam length. The structure is analyzed segment-wise along the beam’s longitudinal length direction. By applying continuity conditions at the interfaces of varying cross-sections and leveraging the structural symmetry, analytical solutions for both the natural frequency and output displacement are derived. The analytical predictions are validated against finite-element results, and experiments also verify the accuracy of the analytical solution of the analytical voltage–displacement response. In addition, the effects of key geometric parameters on the dynamic performance are systematically investigated. The proposed model provides theoretical guidance for tuning the resonance characteristics and drive displacement design of the PMP actuators.

## 1. Introduction

Piezoelectric materials, known for their high precision and nano-scale resolution, are widely used in sensors, energy harvesters, and actuators. Leveraging the inverse piezoelectric effect, actuators have found increasing use in high-precision instruments, such as atomic force microscopes, asymmetric piezoelectric clamps, micro-speakers, and deformable mirrors [1,2,3,4]. A key requirement of these instruments is the ability to achieve micro- to nano-level precision in actuation performance.

The structural design of piezoelectric actuators directly affects their functional adaptability. At present, four mainstream forms of plate, beam, film, and shell have been formed. Different structures show targeted advantages in specific scenarios due to differences in mechanical properties. Among them, piezoelectric plates are often integrated into micro-pump systems in the form of unimorph actuators [5]. During operation, basic deformation is generated by the inverse piezoelectric effect, and then transformed into bending motion to meet the needs of the pump body through the mechanical displacement amplification mechanism to achieve accurate fluid transport. In the innovative applications of other structures, researchers have also achieved remarkable results. For example, Choi et al. [6] developed a three-degree-of-freedom planar actuator based on the high response characteristics of lead zirconate titanate (PZT) film, which greatly improved the motion control dimension of precision equipment. Yuan et al. [7] adjusted the nano-tip deflection sensitivity to a practical level by optimizing the parameters of a ZnO cantilever beam, which provided key support for the improvement of the accuracy of equipment such as atomic force microscope. Zhao et al. [8] and Lian et al. [9] also expanded the improved first-order piezoelectric plate theory from the theoretical level. The regulatory mechanism of piezoelectric thin plates as sensors is explored to provide the theoretical basis for structural design and performance optimization. These technological innovations around different structures not only solve the accuracy, dimension or regulation problems of specific scenarios, but also improve the response accuracy and energy efficiency of various piezoelectric systems as a whole [10]. Among all such structures, beam-type piezoelectric actuators hold an important position in the fields of precision manufacturing and micro-electromechanical systems, owing to their comprehensive advantages including simple structure, ease of processing and assembly, light weight, fast dynamic response, excellent controllability, and flexible parameter adjustment [11,12,13].

However, traditional single-layer piezoelectric patches often remain limited in addressing complex, multi-objective application demands. Bimorph actuators, which utilize oppositely poled piezoelectric layers to generate differential bending, are commonly used due to their improved deformation characteristics. These actuators have been studied both experimentally [14,15] and theoretically, yet their ability to meet comprehensive requirements remains insufficient. This has driven increased interest in heterogeneously structured actuators, such as multilayer piezoelectric stacks and piezoelectric–elastomer hybrid laminates [16]. In particular, sandwich-type actuators featuring localized piezoelectric actuation have shown significant potential for enhancing output performance [17].

Theoretical modeling plays a fundamental role in performance prediction and optimization. Therefore, a wide range of theoretical studies have been conducted on piezoelectric actuators. Aldraihem and Khdeir [18] proposed analytical models for beams integrated with thickness-shear and extension-mode piezoelectric actuators, using both first-order and higher-order beam theories. Majeed et al. [19] provided unified solutions for moderately thick beams with surface-bonded piezoelectric patches using distributed transfer functions. Schoeftner [20] derived closed-form solutions for cantilevered piezoelectric beam deflections under multiphase loading using Timoshenko beam theory. Other studies have also examined monocrystalline and bimorph structures [21], with methods developed to optimize control algorithms for cantilevered sandwich beam configurations.

The precision and dynamic performance of piezoelectric actuators are critical metrics in engineering applications. Several studies have addressed their dynamic characteristics through theoretical and experimental means. Saravanos et al. [22] employed laminated plate theory and the finite element method (FEM) to model displacement and electric potential fields in composite piezoelectric plates for dynamic analysis. Chen et al. [23] explored the dynamic stability of laminated piezoelectric beams under periodic axial loading and implemented active control using negative velocity feedback. Rechdaoui et al. [24] optimized nonlinear dynamic modeling by introducing higher-order multiscale approaches, overcoming the limitations of classical models that neglect quadratic nonlinearities. These studies collectively provide a theoretical basis for dynamic control and for piezoelectric composite structures operating under complex conditions.

Among various novel actuator configurations, the partially laminated piezoelectric–metal–piezoelectric (PMP) beam offers enhanced output displacement and tunability. This work develops an analytical model for a partially laminated PMP actuator by incorporating the piezoelectric constitutive relations, electric potential distribution, and segment-wise continuity conditions. Based on this framework, a segmented model for a variable-cross-section PMP beam is established, and analytical solutions for both the natural frequencies and displacement response are obtained. Finally, the effects of key geometric parameters are systematically examined. In contrast to existing segmented or patch-based beam models, the presented analytical models can simultaneously predict its natural frequencies and output displacement to provide theoretical guidance for resonance tuning and actuator design.

## 2. Theoretical Model

**Figure 1:**
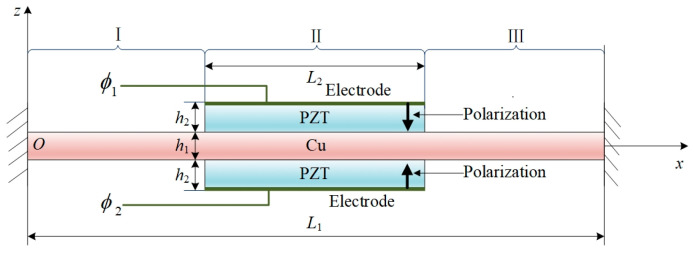
Schematic illustration of the model for a partially laminated PMP actuator. Reprinted with permission from [25]. Copyright (2026) by Springer Nature. Regions I and III denote the metal layer segments on the left and right sides, respectively. Whereas Region II denotes the partially laminated piezoelectric layers attached to the metal substrate.

As illustrated in Figure 1, a partially laminated PMP actuator model is considered. The actuator consists of a central metal layer that serves both elastic and conductive functions and is clamped at both ends. The piezoelectric layers are symmetrically bonded to the upper and lower surfaces of the metal substrate, forming a partial coverage configuration relative to the beam length. Perfect bonding condition is assumed between the piezoelectric and metal layers, with no relative slip at the interfaces, while neglecting the influence of adhesive compliance and shear-lag transfer. The upper and lower surfaces of the structure are each coated with uniform electrodes.

The metal layer has a length *L*_1_ and thickness *h*_1_, while the PZT layers have a length *L*_2_ and thickness *h*_2_. A global coordinate system *oxz* is established, with the origin located at the centroid of the left-end cross-section. The piezoelectric layers are oppositely polarized, where the upper layer is polarized along the negative *z*-axis, and the lower layer is along the positive *z*-axis. Electrodes are bonded to the outer surfaces of the piezoelectric layers. The thickness of the electrodes is much smaller than that of the actuator, and their mechanical contributions are also neglected. Consequently, the electrodes only influence the electric boundary conditions.

For the piezoelectric material layer, the three-dimensional constitutive equations are

(1a) $$\begin{array}{l} \sigma_{xx}^{p}=c_{11}\varepsilon_{xx}^{p}+c_{12}\varepsilon_{yy}^{p}+c_{13}\varepsilon_{zz}^{p}-e_{31}E_{z}^{p},\\ \sigma_{yy}^{p}=c_{12}\varepsilon_{xx}^{p}+c_{11}\varepsilon_{yy}^{p}+c_{13}\varepsilon_{zz}^{p}-e_{31}E_{z}^{p},\\ \sigma_{zz}^{p}=c_{13}\varepsilon_{xx}^{p}+c_{13}\varepsilon_{yy}^{p}+c_{33}\varepsilon_{zz}^{p}-e_{33}E_{z}^{p},\\ \sigma_{yz}^{p}=2c_{44}\varepsilon_{yz}^{p}-e_{15}E_{y}^{p},\\ \sigma_{zx}^{p}=2c_{55}\varepsilon_{xz}^{p}-e_{15}E_{x}^{p},\\ \sigma_{xy}^{p}=2c_{66}\varepsilon_{xy}^{p}, \end{array}$$

(1b) $$\begin{array}{l} D_{x}=2e_{15}\varepsilon_{xz}^{p}+\kappa_{11}E_{x}^{p},\\ D_{y}=2e_{15}\varepsilon_{yz}^{p}+\kappa_{11}E_{y}^{p},\\ D_{z}=e_{31}(\varepsilon_{xx}^{p}+\varepsilon_{yy}^{p})+e_{33}\varepsilon_{zz}^{p}+\kappa_{33}E_{z}^{p}, \end{array}$$

where the superscript ‘*p*’ refers specifically to properties of the piezoelectric layer. *σ_ij_* denotes the stress, *ε_ij_* is the corresponding strain, and *D_i_* is the electric displacement. The parameters *c_ij_* and *e_ij_*_,_ and *κ_ij_* represent the material constants of elastic stiffness, piezoelectric coupling, and dielectric permittivity, respectively. *E_i_* denotes the electric field intensities in different directions.

The corresponding geometrical relationships for the piezoelectric layer are

(2) $$\varepsilon_{xx}^{p}=\frac{\partial u^{p}}{\partial x},\;\varepsilon_{xz}^{p}=\frac{1}{2}\left(\frac{\partial u^{p}}{\partial z}+\frac{\partial w^{p}}{\partial x}\right),\;E_{z}^{p}=-\frac{\partial \phi }{\partial z},\;E_{x}^{p}=-\frac{\partial \phi }{\partial x},$$

where *u* and *w* are the displacements along *x*- and *z*-axes, and *ϕ* represents the electric potential.

As the overall thickness of the actuator is small, the actuator is modeled as a slender laminated beam. The Euler–Bernoulli beam theory is adopted to obtain a concise analytical solution. Therefore, the following plane stress assumptions are introduced for the stress components in both the piezoelectric and elastic layers, that is

(3) $$\sigma_{yy}^{p}=\sigma_{zz}^{p}=\sigma_{yz}^{p}=\sigma_{xy}^{p}=0.$$

By substituting the assumptions into Equation (1), the material equations for the piezoelectric layer can be simplified as

(4) $$\begin{array}{l} \sigma_{xx}^{p}=c_{11}^{p}\varepsilon_{xx}^{p}-e_{31}^{p}E_{z}^{p},\\ \sigma_{zx}^{p}=2c_{44}^{p}\varepsilon_{xz}^{p}-e_{15}^{p}E_{x}^{p},\\ D_{x}=2e_{15}^{p}\varepsilon_{xz}^{p}+\kappa_{11}^{p}E_{x}^{p},\\ D_{z}=e_{31}^{p}\varepsilon_{xx}^{p}+\kappa_{33}^{p}E_{z}^{p}, \end{array}$$

where the derived equivalent material constants are expressed by

(5) $$\begin{array}{c} c_{11}^{p}=\frac{\left(c_{11}-c_{12})(c_{11}c_{33}-2c_{13}^{2}+c_{12}c_{33}\right)}{\left(c_{11}c_{33}-c_{13}^{2}\right)},\\ e_{31}^{p}=\frac{\left(c_{11}-c_{12})(c_{13}e_{33}-c_{33}e_{31}\right)}{\left(c_{13}^{2}-c_{11}c_{33}\right)},\\ c_{44}^{p}=c_{44},\;\;e_{15}^{p}=e_{15},\;\;\kappa_{11}^{p}=\kappa_{11},\\ \kappa_{33}^{p}=\frac{c_{11}^{2}e_{33}^{2}-c_{11}c_{13}e_{31}^{2}-c_{11}c_{13}e_{31}e_{33}+c_{13}^{2}e_{31}e_{33}+c_{11}c_{33}e_{31}^{2}-c_{13}^{2}e_{31}^{2}}{c_{11}(c_{11}c_{33}-c_{13}^{2})}+\kappa_{33}. \end{array}$$

Equation (4) is one-dimensional constitutive equations within the Euler–Bernoulli framework. It should be noted that this derivation process is based on three-dimensional elasticity by introducing the assumption of planar cross-section and simplifying the model through the use of plane stress state.

The corresponding constitutive equations for the elastic layer are obtained as

(6) $$\sigma_{xx}^{e}=E\varepsilon_{xx}^{e},\;\;\sigma_{zx}^{e}=G\varepsilon_{zx}^{e},$$

where the superscript ‘*e*’ denotes the parameters associated with the elastic layer, while *E* and *G* are Young’s modulus and shear modulus, respectively. The corresponding geometrical relationships for the elastic layer are given by

(7) $$\varepsilon_{xx}^{e}=\frac{\partial u^{e}}{\partial x},\;\varepsilon_{zx}^{e}=\frac{1}{2}\left(\frac{\partial u^{e}}{\partial z}+\frac{\partial w^{e}}{\partial x}\right).$$

To provide an additional quantitative measure of electromechanical conversion efficiency, the effective electromechanical coupling coefficient *k_em_* is defined as

(8) $$k_{em}=\sqrt{\frac{U_{m}}{U_{m}+U_{e}}},$$

where $U_{m}$ is the elastic strain energy and $U_{e}$ is the electric energy obtained from the numerical model under the same loading condition, which can be expressed as

(9) $$U_{m}=\int_{L_{1}}\frac{1}{2}\sigma_{xx}^{e}\varepsilon_{xx}^{e}dL_{1},\;U_{e}=\int_{L_{2}}\frac{1}{2}\left(\sigma_{xx}^{p}\varepsilon_{xx}^{p}+D_{x}E_{x}^{p}\right)dL_{2}.$$

## 3. Analytical Solution for Natural Frequency and Dynamic Displacement

According to the working principle of piezoelectric actuators, the voltage is applied to the surface electrodes of the piezoelectric layer, where the electric potential on the upper surface (*z* = *h*_1_/2 + *h*_2_) is set as *ϕ*_1_(t), and on the lower surface (*z* = −*h*_1_/2 − *h*_2_) as *ϕ*_0_(t). The uniform electrode in Figure 1 acting as a conductor, form equipotential plane. Therefore, the electric potential distribution is described using the parallel-plate assumption, which neglects the influence of the edge fringing fields and the converse piezoelectric effect non-uniformity. Therefore, the electric potential distribution within each part of the piezoelectric layer can be expressed as

(10) $$\begin{array}{cc} \phi (z,t)=\frac{\phi_{1}(t)+\phi_{0}(t)}{2}+\left(z-\frac{h_{1}}{2}\right)\frac{\phi_{1}(t)-\phi_{0}(t)}{2h_{2}}, & \;\mathrm{at}\;z\in \left(\frac{h_{1}}{2},h_{2}+\frac{h_{1}}{2}\right),\\ \phi (z,t)=\frac{\phi_{1}(t)+\phi_{0}(t)}{2}, & \;\mathrm{at}\;z\in \left(-\frac{h_{1}}{2},\frac{h_{1}}{2}\right),\\ \phi (z,t)=\phi_{0}(t)+\left(z+\frac{h_{1}}{2}+h_{2}\right)\frac{\phi_{1}(t)-\phi_{0}(t)}{2h_{2}}, & \;\mathrm{at}\;z\in \left(-\left(h_{2}+\frac{h_{1}}{2}\right),-\frac{h_{1}}{2}\right). \end{array}$$

The structure under consideration is a variable-section laminated beam. The process is divided into three parts, covering the intervals: Part I for $x\in \left(0,\left(L_{1}-L_{2}\right)/2\right)$; Part II for $x\in \left(\left(L_{1}-L_{2}\right)/2,\left(L_{1}+L_{2}\right)/2\right)$; Part III for $x\in \left(\left(L_{1}+L_{2}\right)/2,L_{1}\right)$. Based on the Euler-Bernoulli beam theory, the displacement can be expanded along the beam’s longitudinal direction, as given in Part I:

(11a) $$u_{1}(x,z,t)=u_{1}^{\left(0\right)}(x,t)-z\frac{\partial w_{1}(x,t)}{\partial x},$$

for Part II:

(11b) $$u_{2}(x,z,t)=u_{2}^{\left(0\right)}(x,t)-z\frac{\partial w_{2}(x,t)}{\partial x},$$

for Part III:

(11c) $$u_{3}(x,z,t)=u_{3}^{\left(0\right)}(x,t)-z\frac{\partial w_{3}(x,t)}{\partial x},$$

where *u_i_* and *w_i_* represent the displacements of each segment along *x*- and *z*-axes, *u*^(0)^ is the displacement of a point on the reference plane of a laminated structure.

Neglecting the effects of damping, and based on the Euler–Bernoulli kinematics and the sectional force equilibrium of each segment, the governing equation for transverse vibration can be written in the classical beam form [26] as

(12) $${\left(EI\right)}_{j}\frac{\partial^{4}w_{j}}{\partial x^{4}}+{\left(\rho A\right)}_{j}\frac{\partial^{2}w_{j}}{\partial t^{2}}=0,$$

where *w_j_* represents the corresponding displacements of segmented sections, and *j* = 1, 2, 3. *ρ* is the density, and *A* is the cross-sectional area, defined by

(13) $${\left(\rho A\right)}_{1}=\rho_{1}h_{1}(l_{1}-l_{2})/2,\;\;{\left(\rho A\right)}_{2}=\rho_{1}h_{1}l_{2}+2\rho_{2}h_{2}l_{2},\;\;{\left(\rho A\right)}_{3}=\rho_{1}h_{1}(l_{1}-l_{2})/2,$$

where *ρ*_1_ and *ρ*_2_ are the densities of the elastic and piezoelectric layers, respectively. The dynamic displacement is assumed to be in the form

(14) $$w_{j}(x,t)=m_{j}(x)e^{i\omega t},$$

where *m_j_* (*x*) is the spatial displacement function associated with the applied excitation, and *ω* is the angular frequency.

Substituting Equation (14) into Equation (12), the governing equations of motion for each structural segment are obtained as

(15) $$\frac{\partial^{4}m_{j}(x)}{\partial x^{4}}-\gamma_{j}^{4}m_{j}(x)=0,$$

where

(16) $$\gamma_{1}^{4}=\frac{12{\left(\rho A\right)}_{1}\omega^{2}}{Eh_{1}^{3}},\;\;\gamma_{2}^{4}=\frac{12{\left(\rho A\right)}_{2}\omega^{2}}{Eh_{1}^{3}+c_{11}^{p}\left(8h_{2}^{3}+6h_{1}^{2}h_{2}+12h_{1}h_{2}^{2}\right)},\;\;\gamma_{3}^{4}=\frac{12{\left(\rho A\right)}_{3}\omega^{2}}{Eh_{1}^{3}}.$$

The general solution of the differential equation in Equation (15) can be assumed as

(17) $$\begin{array}{c} m_{1}(x)=C_{1}e^{\gamma_{1}x}+C_{2}e^{-\gamma_{1}x}+C_{3}\cos\left(\gamma_{1}x\right)+C_{4}\sin\left(\gamma_{1}x\right),\\ m_{2}(x)=C_{5}e^{\gamma_{2}x}+C_{6}e^{-\gamma_{2}x}+C_{7}\cos\left(\gamma_{2}x\right)+C_{8}\sin\left(\gamma_{2}x\right),\\ m_{3}(x)=C_{9}e^{\gamma_{3}x}+C_{10}e^{-\gamma_{3}x}+C_{11}\cos\left(\gamma_{3}x\right)+C_{12}\sin\left(\gamma_{3}x\right), \end{array}$$

where *C_i_* (*i* = 1, 2, …, 12) are constant coefficients determined by the boundary and continuity conditions.

The structural axial force *N_j_*, bending moment *M_j_*, and shear force *Q_j_* are calculated for each segment, for Part I

(18a) $$N_{1}=\int_{-h_{1}/2}^{h_{1}/2}\sigma_{xx1}dz,\;M_{1}=\int_{-h_{1}/2}^{h_{1}/2}\sigma_{xx1}zdz,\;Q_{1}=\int_{-h_{1}/2}^{h_{1}/2}\sigma_{zx1}dz,$$

for Part II

(18b) $$N_{2}=\int_{-h_{1}/2}^{h_{1}/2}\sigma_{xx2}dz+2\int_{h_{1}/2}^{(h_{1}+2h_{2})/2}\sigma_{xx2}dz,\;M_{2}=\int_{-h_{1}/2}^{h_{1}/2}\sigma_{xx2}zdz+2\int_{h_{1}/2}^{(h_{1}+2h_{2})/2}\sigma_{xx2}zdz,\;Q_{2}=\int_{-h_{1}/2}^{h_{1}/2}\sigma_{zx2}dz+2\int_{h_{1}/2}^{(h_{1}+2h_{2})/2}\sigma_{zx2}dz,$$

for Part III

(18c) $$N_{1}=\int_{-h_{1}/2}^{h_{1}/2}\sigma_{xx3}dz,\;M_{1}=\int_{-h_{1}/2}^{h_{1}/2}\sigma_{xx3}zdz,\;Q_{1}=\int_{-h_{1}/2}^{h_{1}/2}\sigma_{zx3}dz,$$

where *σ_xxi_* and *σ_zxi_* (*i* = 1, 2, 3) denote the stresses of each segment.

Because of the symmetric lay-up and the symmetric bending-type actuation, based on the force balance of each segment, the resultant axial force in each segment is taken to be zero, *N_i_* = 0. The boundary conditions at both ends of the beam are given by

(19) $$\begin{array}{c} w_{1}=0,\;\frac{\partial w_{1}}{\partial x}=0,\;\;\;\mathrm{at}\;x=0,\;\\ w_{3}=0,\;\frac{\partial w_{3}}{\partial x}=0,\;\;\;\mathrm{at}\;x=L_{1}. \end{array}$$

Continuity conditions at the interfaces between segments are specified as

(20) $$\begin{array}{l} w_{1}=w_{2},\;\frac{\partial w_{1}}{\partial x}=\frac{\partial w_{2}}{\partial x},\;M_{1}=M_{2},\;Q_{1}=Q_{2},\;\;\;\mathrm{at}\;x=\frac{L_{1}-L_{2}}{2},\;\\ \;w_{2}=w_{3},\;\frac{\partial w_{2}}{\partial x}=\frac{\partial w_{3}}{\partial x},\;M_{2}=M_{3},\;\;Q_{2}=Q_{3},\;\;\;\mathrm{at}\;x=\frac{L_{1}+L_{2}}{2}. \end{array}$$

It should be noted that in the present segmented formulation, the interfacial conditions require only the mechanical field variables. The corresponding influence of the piezoelectric effect is incorporated through the constitutive relations and the applied electric potentials.

The four boundary conditions in Equation (19), together with the eight continuity conditions in Equation (20), form a system of 12 equations. These equations are used to solve the 12 unknown constant coefficients in Equation (17). The coefficients associated with the unknowns are assembled into a 12 × 12 determinant |*H*|, that is

(21) $$\left|H\right|=0.$$

Solving Equation (21) yields the natural angular frequencies *ω_n_* of the structure for various modes. The corresponding natural frequencies *ƒ_n_* can then be derived as

(22) $$f_{n}=\frac{\omega_{n}}{2\pi }.$$

With the natural frequencies known, the output displacement can subsequently be obtained, for Part I:

(23a) $$w_{1}=C_{1}\left(\phi ,t\right)e^{\gamma_{1}x+i\omega_{n}t}+C_{2}\left(\phi ,t\right)e^{-\gamma_{1}x+i\omega_{n}t}+C_{3}\left(\phi ,t\right)\cos\left(\gamma_{1}x\right)e^{i\omega_{n}t}+C_{4}\left(\phi ,t\right)\sin\left(\gamma_{1}x\right)e^{i\omega_{n}t},$$

for Part II:

(23b) $$w_{2}=C_{5}\left(\phi ,t\right)e^{\gamma_{2}x+i\omega_{n}t}+C_{6}\left(\phi ,t\right)e^{-\gamma_{2}x+i\omega_{n}t}+C_{7}\left(\phi ,t\right)\cos\left(\gamma_{2}x\right)e^{i\omega_{n}t}+C_{8}\left(\phi ,t\right)\sin\left(\gamma_{2}x\right)e^{i\omega_{n}t},$$

for Part III:

(23c) $$w_{3}=C_{9}\left(\phi ,t\right)e^{\gamma_{3}x+i\omega_{n}t}+C_{10}\left(\phi ,t\right)e^{-\gamma_{3}x+i\omega_{n}t}+C_{11}\left(\phi ,t\right)\cos\left(\gamma_{3}x\right)e^{i\omega_{n}t}+C_{12}\left(\phi ,t\right)\sin\left(\gamma_{3}x\right)e^{i\omega_{n}t}.$$

## 4. Verification of Numerical and Experimental Results

Considering a constrained space of 10 mm as the design limit, the different piezoelectric layer lengths are established. The elastic layer is made of Cu with Young’s modulus *E* = 127 GPa, serving as a pure conductor. The piezoelectric layers are composed of PZT-5H, with material constants listed in Table 1.

### 4.1. Numerical Validation

Finite-element simulations were implemented within the COMSOL Multiphysics (version 5.3a) using the Solid Mechanics and Electrostatic modules. The metallic and piezoelectric layers were discretized using quadrilateral elements, and the full electromechanical coupling inherent to the PZT layers was rigorously incorporated. Clamped boundary conditions were enforced at both ends of the beam structure. A systematic mesh convergence analysis was conducted to ensure numerical reliability. In the adopted converged mesh, the maximum element dimension was specified as 0.02 mm, yielding a total element count of 65,000. Successive mesh refinement was shown to induce negligible variations in the first two natural frequencies, thereby validating mesh convergence.

**Figure 2:**
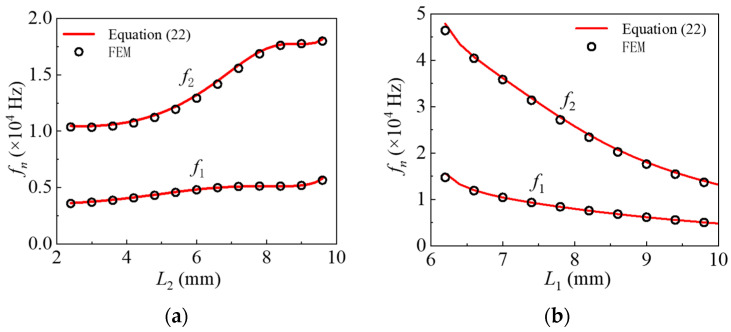
Comparison of analytical solutions and numerical results for natural frequencies: (**a**) under varying *L*_1_; (**b**) under varying *L*_2_.

A piezoelectric layer with a fixed length of *L*_2_ = 6 mm is used, while the length of the elastic layer *L*_1_ is varied from 6 mm to 10 mm for modeling and analysis. The resulting natural frequencies of the actuator structure are presented in Figure 2a. The maximum deviation between the theoretical predictions and finite element analysis results is 2.45%, confirming the accuracy of the analytical model. The natural frequencies of the structure decrease as the length of the elastic layer increases. When the elastic layer is relatively short, both the first- and second-order natural frequencies exhibit significant variation. However, as the elastic layer approaches the maximum spatial limit, the rate of frequency change diminishes. For dynamic actuation applications, it is recommended to design the structure such that the first- and second-order natural frequencies are close to each other. This configuration allows for more flexible tuning of the applied driving signal to match the actuator’s dynamic characteristics.

The overall length of the structure model is *L*_1_ = 10 mm, while the length of the piezoelectric layer *L*_2_ is varied from 2 mm to 10 mm. The comparison in Figure 2b shows a maximum discrepancy of only 2.18%, demonstrating strong agreement and validating the accuracy of the theoretical model. When the length of the piezoelectric segment is small, both the first- and second-order natural frequencies exhibit a slight increasing trend. As the piezoelectric length increases, the second-order natural frequency undergoes more noticeable variation. However, when the length of the piezoelectric layer exceeds 8 mm, the natural frequency gradually stabilizes, indicating that within this range, the influence of the piezoelectric layer length on the natural frequency gradually weakens. These observations offer valuable guidance for structural dimensional optimization in dynamic actuation applications and enable frequency-specific control of actuation performance.

The overall length of the structure model is *L*_1_ = 10 mm with *h*_1_ = 0.1 mm, the piezoelectric layer with *L*_2_ = 8 mm yield the effective electromechanical coupling coefficient *k_em_* = 0.7449 based on Equation (8). While, for a 6 mm piezoelectric layer, *k_em_* = 0.7496. This partially laminated PMP actuator results in a relatively small difference in the effective electromechanical coupling coefficients between the two cases, both of which are all much higher than those of typical dual-chip systems. Therefore, this configuration is more conducive to enhancing the electromechanical response strength, control efficiency and energy conversion capability of piezoelectric semiconductor devices.

### 4.2. Experimental Validation

In the theoretical analysis presented in Section 2 and Section 3, the governing equations for piezoelectric materials and elastic materials are linearized. Consequently, the normalized driving displacements and natural frequency are independent of the material size. To verify the correctness of the analytical solution of the voltage–displacement response, a cheap large sample was purchased to measure and verify, where 60 mm is in length and 40 mm in width, with a thickness of 0.2 mm. The piezoelectric material PZT-5H is used in the experiment.

A digital laser, Doppler vibrometer (D-VD-5-24, Polytec GmbH, Waldbronn, Germany), is employed to measure the dynamic driving displacement of the piezoelectric laminated beam structure. In engineering applications, sinusoidal voltage signals are commonly used to drive, enabling smooth and precise motion. Therefore, a sine wave signal was generated by the Agilent 33220A (Agilent Technologies, Santa Clara, CA, USA) function generator with an excitation frequency of 500 Hz, as

(24) $$\phi (t)=V_{0}\sin(\omega t).$$

where *V*_0_ denotes the voltage amplitude. In the present experiments, the applied peak-to-peak voltages were 1 V, 4 V, and 10 V, corresponding to voltage amplitudes of 0.5 V, 2 V, and 5 V, respectively.

**Figure 3:**
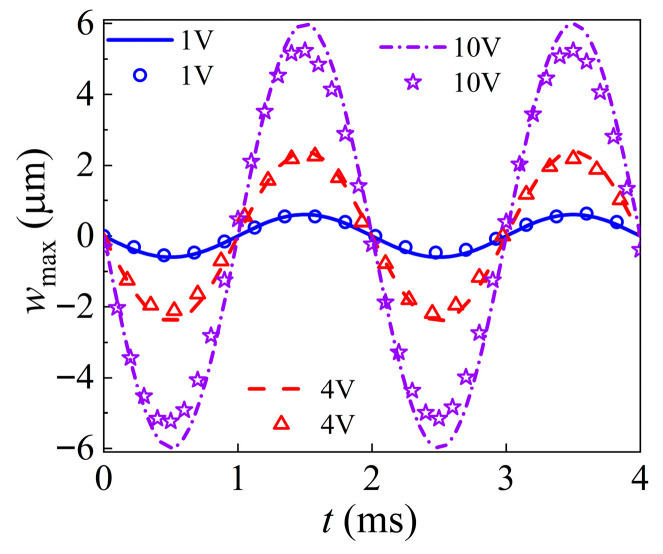
Comparison between the experimentally measured and analytically predicted displacement responses under different voltage levels at a driving frequency of 500 Hz, where points represent the experimental results, and lines represent the results of Equation (23).

The analytical solutions and experimental results closely match under identical voltage conditions, as shown in Figure 3, with peak and valley timings aligning nearly perfectly. The analytical predictions and experimental results show consistent response trends under the same voltage conditions, providing experimental verification for the proposed theoretical framework.

## 5. Parametric Study and Design Implications

**Figure 4:**
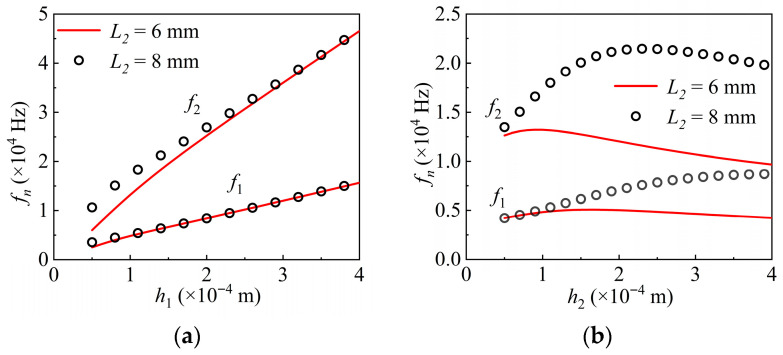
Numerical results of the natural frequencies: (**a**) under different *h*_1_; (**b**) under different *h*_2_.

The overall length of the structure model is *L*_1_ = 10 mm, and two piezoelectric layer configurations, i.e., 6 mm and 8 mm in length with a uniform thickness *h*_2_ = 0.1 mm, are selected to examine the relationship between natural frequencies and the thickness *h*_1_ of the elastic layer, as shown in Figure 4a. As the elastic layer thickness increases, both the first- and second-order natural frequencies rise significantly for both configurations. A thinner elastic layer facilitates resonance excitation under dynamic voltage input, as the resulting increase in natural frequency enhances the likelihood of matching the applied driving frequency with the structure’s resonance conditions.

With the elastic layer thickness fixed at *h*_1_ = 0.1 mm, the influence of piezoelectric layer thickness *h*_2_ on the natural frequencies is shown in Figure 4b. When the piezoelectric layer is short, both the first- and second-order natural frequencies initially increase with increasing thickness, then begin to decrease beyond a critical thickness. In contrast, for a long piezoelectric layer, the first natural frequency increases monotonically with thickness, while the second natural frequency shows a non-monotonic trend, rising at first and then declining. These findings provide a crucial theoretical basis for optimizing geometric parameters, such as the aspect ratio, in the design of piezoelectric actuators.

**Figure 5:**
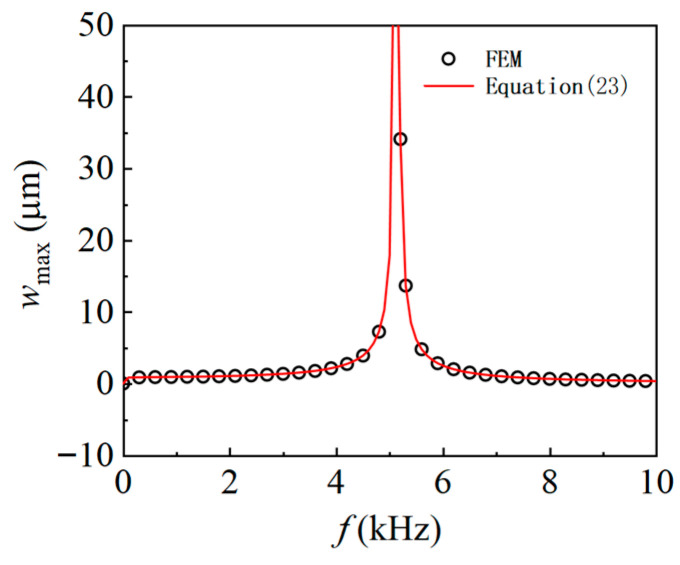
Predicted frequency-response curve of the partially laminated PMP actuator.

For an actuator, the elastic layer length is set to 10 mm, and the piezoelectric layer length is 8 mm, with both layers having a uniform thickness of 0.1 mm. The frequency-response characteristics of the partially laminated PMP actuator were numerically examined, as shown in Figure 5. The displacement magnitude exhibits a pronounced peak near the resonance frequency, indicating strong frequency selectivity of the actuator’s response. Calculations determine the first-order natural frequency of the structure to be 5109 Hz.

**Figure 6:**
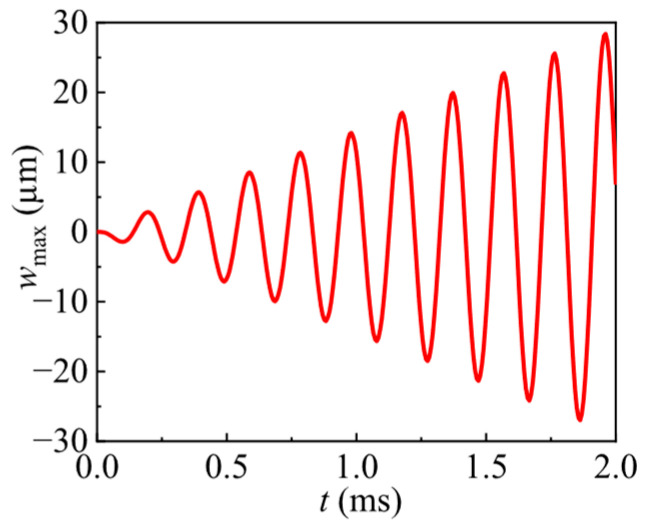
Numerically predicted transient resonant response at the center of the structure under a 5109 Hz sinusoidal voltage excitation.

When a sine wave at this natural frequency is applied in the FEM, the initial transient stage of the dynamic response in the undamped numerical prediction is shown in Figure 6. It shows a significant increase in displacement amplitude under resonant excitation in the undamped numerical prediction. This demonstrates that matching the driving frequency to the structural resonance can significantly enhance the efficiency of piezoelectric actuators. It should be pointed out that the observation window in Figure 6 is limited to the initial transient stage of the numerical response and does not represent the fully developed steady-state resonant motion. In practical applications, the amplitude would be limited by damping and other loss mechanisms.

## 6. Conclusions

In this paper, we have established a dynamic model for a partially laminated PMP actuator based on the Euler–Bernoulli beam theory. The analytical solutions for the natural frequencies and output displacement were obtained. Their accuracy was validated against finite-element results, while experiment measurements further verified the validity of the analytical voltage–displacement response. The main conclusions are summarized as follows:

1. The second-order natural frequency is sensitive to the length of the piezoelectric layer, while the first-order natural frequency remains relatively stable, which enables frequency-specific control of precision actuators.
2. The natural frequencies are sensitive to both the elastic-layer thickness and the piezoelectric-layer thickness. Reducing the elastic-layer thickness and selecting an appropriate piezoelectric-layer thickness provides an effective way to tune the resonance characteristics of the actuator.
3. A significant amplification of displacement occurs when the driving frequency approaches the structural resonance frequency. Matching the driving frequency to the structural resonance can significantly enhance the efficiency of piezoelectric actuators.

It should be pointed out that the present work establishes an analytical model for partially laminated PMP actuators under the ideal elastic conditions with perfect bonding, without considering more complex application conditions, such as the adhesive compliance, the mechanical effects of electrodes, damping and viscoelasticity, fringing electric fields, and material uncertainty. The influence of these complex factors, along with magnitude-phase Bode plots and quality factor identification will be addressed in future studies. Therefore, this paper offers theoretical foundations and design guidelines for structural dimensional optimization in dynamic actuation applications and could enable the frequency control and output displacement control of actuation performance.

## Figures and Tables

**Table 1:** The material properties of piezoelectric layers [25].

Elastic Constant (GPa)	Piezoelectric Constant (C/m^2^)	Dielectric Constant (nF/m)	Density (kg/m^3^)
*c*_11_ = 127.2 *c*_12_ = 80.2	*e*_31_ = −6.6	*κ*_11_ = 15.09	*ρ*_1_ = 8960
*c*_13_ = 84.7 *c*_33_ = 117.4	*e*_33_ = 23.2	*κ*_33_ = 12.69	*ρ*_2_ = 7500
*c*_44_ = 23.0	*e*_15_ = 17.0		

## Data Availability

The data presented in this study are available from the corresponding authors upon request.
